# Fatigue Modeling via Mammalian Auditory System for Prediction of Noise Induced Hearing Loss

**DOI:** 10.1155/2015/753864

**Published:** 2015-11-24

**Authors:** Pengfei Sun, Jun Qin, Kathleen Campbell

**Affiliations:** ^1^Department of Electrical and Computer Engineering, Southern Illinois University, Carbondale, IL 62901, USA; ^2^Department of Surgery, School of Medicine, Southern Illinois University, Springfield, IL 62794, USA

## Abstract

Noise induced hearing loss (NIHL) remains as a severe health problem worldwide. Existing noise metrics and modeling for evaluation of NIHL are limited on prediction of gradually developing NIHL (GDHL) caused by high-level occupational noise. In this study, we proposed two auditory fatigue based models, including equal velocity level (EVL) and complex velocity level (CVL), which combine the high-cycle fatigue theory with the mammalian auditory model, to predict GDHL. The mammalian auditory model is introduced by combining the transfer function of the external-middle ear and the triple-path nonlinear (TRNL) filter to obtain velocities of basilar membrane (BM) in cochlea. The high-cycle fatigue theory is based on the assumption that GDHL can be considered as a process of long-cycle mechanical fatigue failure of organ of Corti. Furthermore, a series of chinchilla experimental data are used to validate the effectiveness of the proposed fatigue models. The regression analysis results show that both proposed fatigue models have high corrections with four hearing loss indices. It indicates that the proposed models can accurately predict hearing loss in chinchilla. Results suggest that the CVL model is more accurate compared to the EVL model on prediction of the auditory risk of exposure to hazardous occupational noise.

## 1. Introduction

Noise induced hearing loss (NIHL) is a serious problem that affects many people worldwide. According to the World Health Organization (WHO), exposure to excessive noise is the major avoidable cause of permanent hearing loss [1]. It is estimated that over 500 million individuals are at risk of developing NIHL [2] globally. Hearing loss has been shown to lower the quality of life, impair social interactions, cause isolation, and even cause loss of cognitive function [3]. As a common public health issue, NIHL has attracted great endeavors devoted to its fundamental mechanism studies. NIHL can be briefly categorized into two types: acoustic trauma caused hearing loss and gradually developing hearing loss (GDHL). Acoustic trauma occurs rapidly and results in an immediate and permanent hearing loss. In acoustic trauma, the inner ear tissue can be stretched beyond its elastic limits by high-level noise in a short duration exposure. For example, an impulse noise with sound pressure level (SPL) above 120 dB could cause acoustic trauma. Unlike acoustic trauma, GDHL is developed over time and can be caused by occupational noise exposure (e.g., low exposures of at least 85 dBA over 8 hours [3]). This type of damage is relative to the noise level and exposure time.

To estimate NIHL, various standards and regulations have been developed over years, for example, ISO 1999:2013 [4], ANSI-S3.44-1996 [5], NOISH98 [6], MIL STD-1474D [7], and CHABA [8]. The noise metrics in these standards and regulations were developed based on either waveform based empirical strategies (e.g., peak acoustic pressure and pulse duration) or auditory weighting based equal energy hypothesis (EEH) (e.g., A-weighted equivalent sound pressure level, *L*_Aeq_) [9–11]. These noise metrics can either characterize the noise exposure or evaluate the acoustic energy of the noise very well, but they do not provide physical insight into the processes of noise induced hearing damage.

In recent years, the mammalian auditory model has been utilized to develop more advanced models for assessment and prediction of NIHL. Price [12] developed an auditory hazard assessment algorithm for humans (AHAAH) model, which was based on theoretical modeling of human auditory transfer functions to investigate the mechanical damage in human ear, caused by high-level impulse noise. The AHAAH model achieved a considerable high accuracy on prediction of impulse noise induced human hearing loss [13]. However, the AHAAH model is only applicable to predict the acoustic trauma induced by high-level impulsive noise (SPL ≥ 140 dB). It cannot estimate GDHL caused by occupational noise (e.g., a Gaussian continuous noise with SPL ≥ 85 dBA). In another study [14], Song applied an analog auditory based model to predict human NIHL. His model used the velocity of the stapes in the middle ear as the input loads but had no involvement with the auditory fatigue theory in the process of hearing damage. Their results showed that Song's model demonstrated a weak correlation with chinchilla NIHL data. In our study, we propose two fatigue models, which combine the mammalian auditory model and high-cycle auditory fatigue theory, for prediction of GDHL caused by occupational noise.

Numerous auditory models (AMs) have been developed in the past decades [16–22]. These models can be categorized into two different groups: functional models and analog models. The functional models are developed based on the observation of input-output behavior of the auditory system with respect to physiological or psychological responses [19]. The functional models usually can achieve high accuracy on prediction of the output of auditory system. The analog models are derived from representing the peripheral ear. Benefited from new advances in auditory physiology, they can explicitly simulate the precise internal physical mechanisms and provide access to all internal physical variables of the underlying analog network models [19].

For NIHL study, the key consideration for choosing an AM is how to accurately quantify the flow of acoustic power from the environment into the inner ear [15]. In addition, the auditory fatigue is strongly correlated with vibration of basilar membrane (BM) in cochlea [23]. It means that an AM is required to provide an accurate estimation of the BM response. Therefore, the functional models focusing on input-output simulation would be more suitable for the investigation of NIHL.

In functional auditory models, three families of auditory filters have been developed, including the rounded exponential (roex) family, the gammatone family (including gammachirp and all-pole and pole-zero variants), and the filter cascades (both all-pole and pole-zero variants) [24]. The roex family is useful mostly as a descriptive model, which describes the shape of magnitude transfer function of an auditory filter [25]. It has no-time domain equivalent and no “runnable” implementation. Comparatively, filter-cascade family minimizes the total computational complexity [26] and demonstrates structural efficiency. As a cascade filter model, triple-path nonlinear (TRNL) filter and its prototype dual resonant nonlinear (DRNL) filter have been proved to be extremely efficient in simulating mammalian auditory system (e.g., human and chinchilla) [27, 28]. Hence, in our study, TRNL filter will be introduced to simulate the transfer function of mammalian inner ear and obtain the BM velocities in cochlea.

In material science, fatigue is a progressive and localized structural damage of a material caused by repeatedly applied loads. There are two kinds of material fatigues, low-cycle and high-cycle fatigues [29]. For low-cycle fatigue, the applied loading is high enough to produce functional material failure in a single cycle or a few cycles. For high-cycle fatigue, the magnitude of stress in each cycle is not sufficient to generate functional material failure with a few cycles. Large numbers of cycles are needed to generate material failure in high-cycle fatigue model.

Assuming that the hearing loss intrinsically is a mechanical failure of the auditory system (i.e., basilar membrane or hair cells) [30–32], NIHL [33] can be regarded as the auditory fatigue [23, 34]. Therefore, the fatigue model based on material theory can be introduced to describe how the noise stimulus causes damage in cochlea in NIHL study. In AHAAH model, the authors used low-cycle fatigue to describe the ear damage [35], in which high-level impulse noise causes the detachment of the organ of Corti from the BM and produces permanent damage in a few noise pulses. For GDHL, the processes of damage in cochlea caused by occupational or environmental noise with SPL from 85 dB to 120 dB [36] can be considered as the high-cycle fatigue [23, 34]. In GDHL, damage in inner ear is the result of repeated flexing of the BM under noise exposure [37], where the outer hair cell (OHC) and the inner hair cell (IHC) are squeezed or stretched leading to hearing loss. Moreover, Moore [33] summarized five major factors influencing the auditory fatigue: (1) the intensity of the noise, (2) the duration of the noise, (3) the frequency of the noise, (4) the recovery interval, and (5) the auditory system's own fatigue frequency (i.e., comparable with material fatigue cycle). Among these factors, intensity, duration, and frequency directly reflect the characteristics of noise and can be applied to develop the auditory fatigue model in our study.

In addition, instead of using the displacement of BM as the loads in [35], the velocity of BM is used as the complex loads on the organ of Corti in our proposed models [38]. The velocity of BM not only reflects the acoustic power flowing into the inner ear, but also highly correlated with strain and loads [37, 39]. Therefore, velocities can be fed into the auditory fatigue model to predict NIHL [40].

In this study, two BM velocities based fatigue models, equivalent velocity level (EVL) and complex velocity level (CVL), are proposed to predict the GDHL caused by occupational noise. The auditory model combining the external-middle ear model and the TRNL filter is applied to obtain the BM velocities at different partitions of cochlea (i.e., Equivalent Rectangular Band (ERB)). Based on the stress against cycles to failure (*S*-*N*) curve and the Miner rule in the high-cycle fatigue theory, the EVL and CVL fatigue based models are developed, respectively. In addition, a series of chinchilla noise exposure data are applied to validate the effectiveness of our developed models for prediction of NIHL in this paper.

## 2. Methods and Materials

### 2.1. Transfer Functions of Chinchilla Auditory System

**Figure 1:**
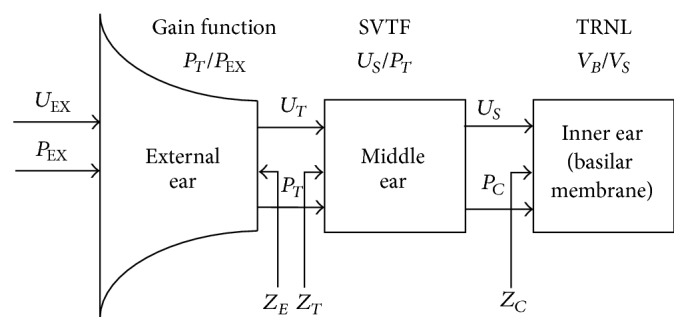
A schematic diagram of a model of auditory periphery, consisting of external ear, middle ear, and inner ear sections [15].

As shown in Figure 1, mammalian ear consists of three sections: external ear, middle ear, and inner ear. The external ear is made up of ear canal, concha, and pinna flange. The middle ear contains tympanic membrane (TM), middle-ear air spaces, eustachian tube, and ossicles. The fluid-filled inner ear contains basilar membrane and organ of Corti [39, 41]. Because the animal noise exposure data used in this study is collected using chinchilla, we developed the proposed fatigue models based on the auditory transfer functions of chinchilla. The ears of other mammals (e.g., human and cat) are highly similar in structure. The same principles used in the modeling can be easily transferred with modest adaptations to fit their anatomic details [39].

#### 2.1.1. External Ear and Middle Ear

The primary function of the external ear and middle ear is gathering sound energy and conducting it into the inner ear. The middle ear acts as an impedance-matching device that extracts acoustic power from the stimulus and transmits it to cochlea [42–45].

**Figure 2:**
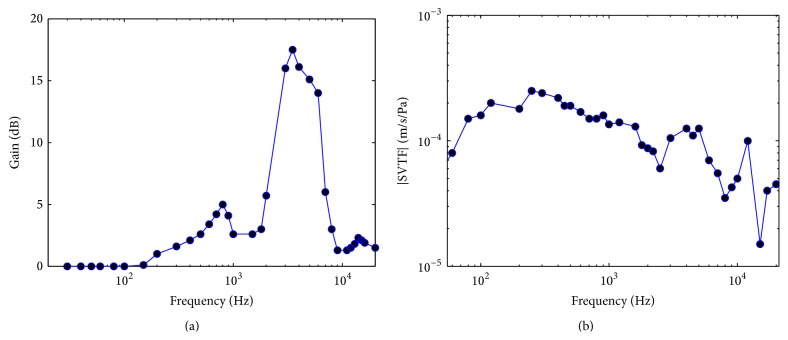
(a) The gain of the external ear and (b) the transfer function of the middle ear of chinchilla.

The primary path for conducting environmental sound into inner ear is through the coupled motion of TM, ossicles, and stapes footplate. One can consider the “ossicular coupling” of sound to inner ear as a cascade of interdependent acoustical and mechanical processes, in which outputs of one stage act as inputs to the next stage. First, the pressure *P*_EX_ and volume velocity *U*_EX_ of noise travel through concha and ear canal and then interact with the TM in the middle ear to produce a pressure *P*_TM_ and volume velocity *U*_TM_ on the TM. Correspondingly, the gain for the external ear can be defined as *G*_*e*_ = *P*_TM_/*P*_EX_, shown in Figure 2(a). Second, the pressure and volume velocity of TM are converted into the pressure *P*_*S*_ and volume velocity *U*_*S*_ acting on stapes in the middle ear, which work against the acoustic impedance of cochlea to produce sound pressure *P*_*C*_ within the cochlea vestibule. In our study, transfer function of the middle ear is characterized by stapes velocity transfer function (SVTF) as shown in Figure 2(b). The SVTF is defined as the ratio between linear velocity of stapes *V*_*S*_ and sound pressure near the TM in the ear canal *P*_TM_ (SVTF = *V*_*S*_/*P*_TM_) [43], where the linear velocity *V*_*S*_ can be obtained through dividing volume velocity *U*_*S*_ by average area of the footplate (*A*_FP_ = 2 mm^2^ [46]).

#### 2.1.2. Inner Ear Model-TRNL Filter

One can assume that cochlea is a two-chambered, fluid-filled box with rigid side walls [35], and the partition between chambers is rigid, except that BM is flexible with elastic deformation. When the stapes motion produces pressure within the cochlea vestibule, sound stimulus can be transferred to OHC and IHC by vibrations of BM [47]. At different sites of BM, which are sensitive to different frequency ranges, the resulting velocities are different. Several phenomenological models have been introduced to simulate the experimental measurements over different sites along BM [27, 48–50].

**Figure 3:**
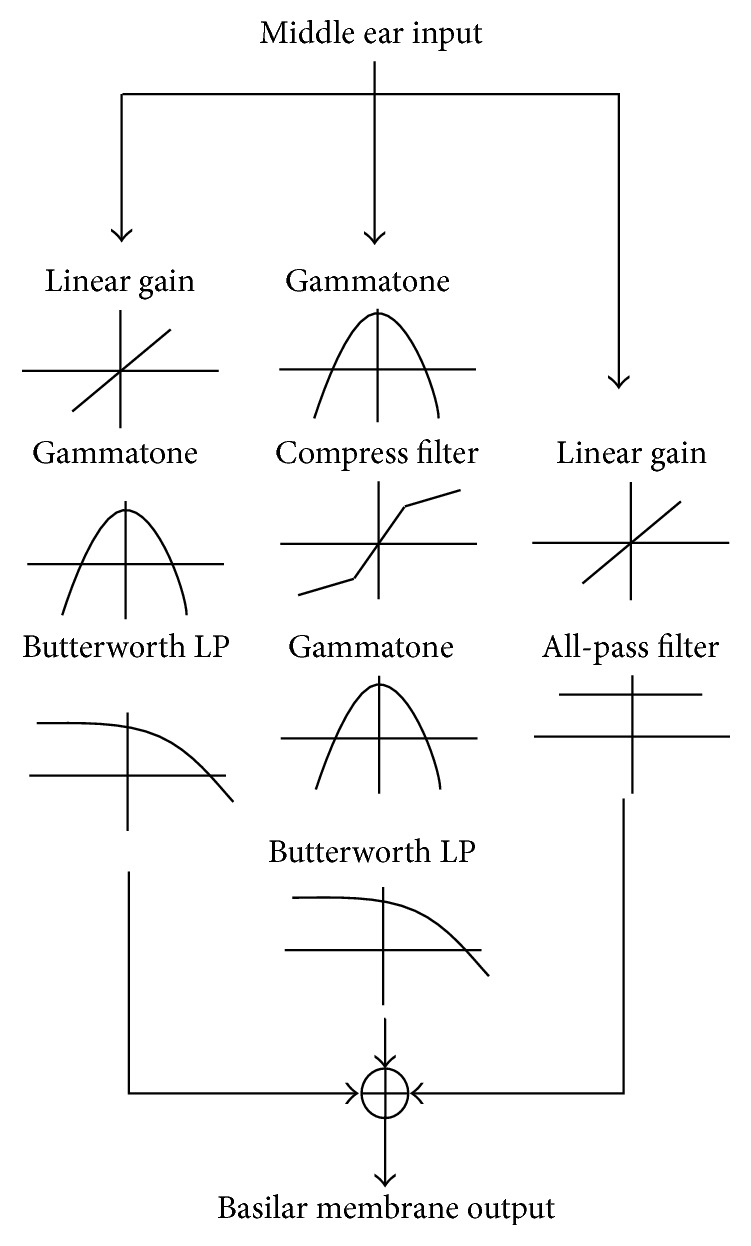
Schematic diagram of the TRNL filter, in which the velocities of stapes in middle ear are passed through three parallel branches to obtain the velocities of BM.

In this study, the TRNL filter introduced in [28] has been utilized to obtain the complex features of BM responses along the partitions of chinchilla's cochlea. The TRNL filter is an improved form of DRNL filter originally proposed in [27]. It can accurately simulate the motion of mammalian BM. As shown in Figure 3, the input for TRNL filter is the linear velocity of stapes *V*_*S*_ and the output represents the BM velocity of a particular location along the cochlea partition. Each individual site is represented as a tuned system including three parallel signal-processes paths: one linear (left), one nonlinear (middle), and one low-gain linear (right). The first linear path consists of a bandpass function, a low pass function, and a gain/attenuation factor, *g*, in a cascade. The nonlinear path is a cascade combination of a bandpass function, a compression function, a second bandpass function, and a low pass function. The third linear path includes a linear, low-gain, all-pass filter. The output of the system is the sum of outputs of the three signal-processing paths.

Each individual bandpass function is a cascade of two or more gammatone filters [51] with unit gain at center frequency (CF). The low pass function consists of second-order low pass filters. In the nonlinear path, the CFs and bandwidths (BW) of the two sets of gammatone filters are the same. In addition, the shape of the compressive function in the nonlinear path is chosen based on animal data and can be defined as

(1) $$\begin{array}{c} y\left[\;t\;\right]=\text{SIGN}\left(\;x\left[\;t\;\right]\;\right)\times \text{MIN}\left(\;a\left|\;x\left[\;t\;\right]\;\right|,b{\left|\;x\left[\;t\;\right]\;\right|}^{c}\;\right), \end{array}$$

where *x*(*t*) is the output of the first filter in the nonlinear path. *a*, *b*, and *c* are parameters of the model. The values for these parameters are summarized in Table 1.

### 2.2. Basilar Membrane Velocity Based Fatigue Models

In this study, two fatigue models, the *S*-*N* curve based EVL model and the Miner rule based CVL model, are proposed to calculate noise induced cumulative hazard, denoted by the number of fatigue cycles.

#### 2.2.1. Equivalent Velocity Level Model

The EVL model is proposed based on the *S*-*N* curve. In this model, the BM velocities, which are regarded as stresses (“*S*”), correspond to the hearing damage, which can be quantitatively denoted by numbers of cycles (“*N*”) that cause the functional failure of hair cells. Assuming that hearing loss level of a deaf ear is 1 and the hearing loss level of a normal ear is 0, we can quantitatively describe the degree of hearing loss in Δ*t* as

(2) $$\begin{array}{c} H_{V\left(t\right),\Delta t}=\frac{\int_{\Delta t}V\left(t\right)dN\left(t\right)}{H_{0}}, \end{array}$$

where *V*(*t*) and *N*(*t*) are the velocity and corresponding failure cycles at time *t*, respectively. Considering that it takes long time to produce GDHL, the *S*-*N* curve used in this study can be considered as a linear approximation. Accordingly, *H*_0_ = *V*_0_ *N*_0_ represents the total hearing loss and can be normalized as 1. Furthermore, supposing that the hearing damage is calculated in unit time period, which means that *dN*(*t*) = 1, and assuming that the negative velocity produces the same level hazard as the positive velocity, *H*_*V*(*t*),Δ*t*_ in (2) can be transformed as

(3) $$\begin{array}{c} H_{V\left(t\right),\Delta t}=\underset{\Delta t}{\sum }V^{2}\left(\;t\;\right). \end{array}$$

Thus, the EVL model can be defined as

(4) Li,EVL=10∗log10⁡∑jVi,j2V02,LI,EVL=10∗log10⁡∑i⊂I∑jVi,j2V02,

where *V*(*i*, *j*) refers to the velocity of the *i*th ERB band of BM at time *j*. The constant *V*_0_ is the BM velocity located at the ERB with 1 kHz center frequency. To characterize the auditory system, ERB based frequency band physically corresponds to the space ERB distance of BM [52]. *L*_*i*,EVL_ reflects the integration of hearing loss level at the *i*th ERB temporally. Comparatively, *L*_*I*,EVL_ is the hearing loss level in frequency band *I*, in which several ERBs might be included. Based on the EVL model, the concept described in (4) can be used to assess the auditory risk of hazard caused by the vibration of BM, which leads to hearing loss.

#### 2.2.2. Complex Velocity Level Model

**Figure 4:**
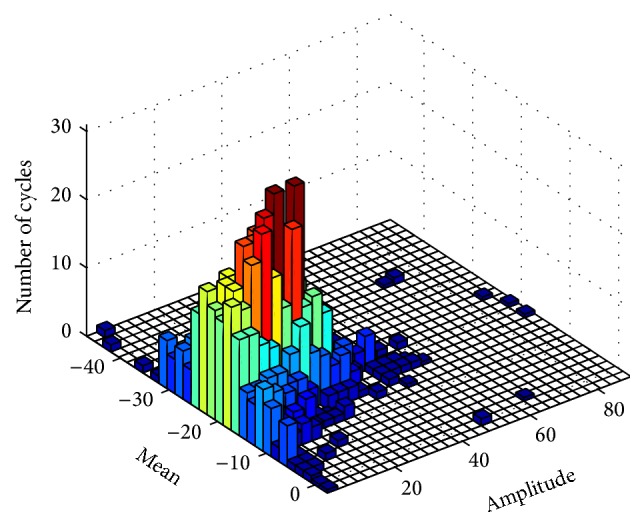
Rainflow matrix of BM velocities at the *i*th ERB in 1 second.

The CVL model is designed based on the Miner rule, which has been commonly used to predict high-cycle fatigue life. In the EVL model, the transition of adjacent stimulus is not accounted for because the loads in the *S*-*N* curve are generated as sinusoidal functions with single frequency. In practice, occupational noise should be considered as complex and often random loads. Obviously, the transition between two adjacent loads can cause more serious NIHL than two individual loads. Therefore, both the amplitude and the transition of loads could significantly affect the life cycle in a fatigue model. The Miner rule includes *S*-*N* relation and also incorporates a new parameter defined as the mean of adjacent loads. For instance, supposing that two adjacent loads with *v*(*t*) = 5 and *v*(*t* + 1) = −6, they will only be characterized according to their amplitudes as 5 and 6 under the *S*-*N* circumstance. In contrast, in the Miner rule, they can be characterized according to both the amplitude and the mean value of the two loads, regarded as |(*v*(*t*) + *v*(*t* + 1))/2|, which will be applied to assess the corresponding damage. In order to obtain the amplitude and mean value distribution of the input, rainflow counting algorithm is developed, which is used to reduce a spectrum of varying stress into a set of simple stress reversals [53]. The concept of the Miner rule based on rainflow algorithm has been illustrated in Figure 4.

As shown in Figure 4, the histogram of loads with different amplitudes and mean values are obtained at *i*th ERB in certain time duration and can be described as

(5) $$\begin{array}{c} B\left(\;\frac{v_{1}}{V_{1}}+\frac{v_{2}}{V_{2}}+\cdots +\frac{v_{k}}{V_{k}}\;\right)=1, \end{array}$$

where *B* is a constant and can be treated as unit. *v*_*k*_ is the number of cycles of load in *k*th category with a *V*_*k*_ failure cycles. The number of categories is defined as *K* = *N*_Amplitude_ *∗N*_Mean_, where *N*_Amplitude_ and *N*_Mean_ are the length of the *x*-axis and *y*-axis in Figure 4. Based on the histogram of complex loads (i.e., velocities), with respect to both amplitudes and mean values, the hearing loss *H*_*i*,CVL_ is the integration of different types of inputs along time axis as follows:

(6) $$\begin{array}{c} H_{i,\text{CVL}}=\underset{j^{\prime }\subset K}{\sum }N_{j^{\prime }}*A\left(\;i,j^{\prime }\;\right)*M\left(\;i,j^{\prime }\;\right), \end{array}$$

where *A*(*i*, *j*′) is the amplitude of velocity and *M*(*i*, *j*′) is the mean value of the adjacent velocities. Based on the *i*th ERB hearing loss *H*_*i*,CVL_, CVL can be obtained:

(7) Li,CVL=10∗log10⁡Hi,CVL2H02,LI,CVL=10∗log10⁡∑i⊂IHi,CVL2H02,

where *H*_0_ is the hearing loss at the ERB with 1 kHz CF. Similar to the EVL model, *L*_*i*,CVL_ and *L*_*I*,CVL_ are the log scale metrics of hearing loss at *i*th ERB and *I* frequency band.

### 2.3. Experimental Data

A series of the animal experimental data were used for effectiveness validation of the proposed fatigue models. The animal data were provided by the research group at State University of New York at Plattsburgh and were used in published animal noise exposure experiments [54, 55]. 22 noise samples were designed, including 3 Gaussian continuous noises with 90, 95, and 100 dBA, respectively, and 19 complex noises (one at 95 dBA, two at 90 dBA, and 16 at 100 dBA). The complex noises were generated by combining different forms of impulse noise with a Gaussian continuous noise [54, 55]. The digitally recorded noise samples (320 sec for each noise sample), which have been applied in the animal exposure experiments [56], are used for noise analysis in this study.

Detailed descriptions of the noise data and experimental protocols of animal studies are available in various publications [54, 55]. In the animal noise exposure experiments, 273 chinchillas in 22 groups were exposed to 22 different noises in five consecutive days and 24 hours per day and then were allowed to recover for 30 days. Auditory evoked potential (AEP) before exposure (PRE), auditory evoked potential after exposure (TS0), and auditory evoked potential after 30 days after exposure (TS30) were measured at 0.5, 1, 2, 4, 8, and 16 kHz for each animal. Both permanent threshold shift (PTS) and temporary threshold shift (TTS) were determined based on the AEP data. From the perspective of physical damage, percentage of outer hair cell loss (%OHC) and percentage of inner hair cell loss (%IHC) were also obtained. In addition, to assess the hearing loss in whole frequency band, the averaged effective PTS has been proposed as PTS_1248_ = (1/4)(PTS_1_ + PTS_2_ + PTS_4_ + PTS_8_), where PTS_1_, PTS_2_, PTS_4_, and PTS_8_ are the PTS values measured at the one-octave band with center frequencies 1, 2, 4, and 8 kHz, respectively. The same rule is applied to TTS to obtain TTS_1248_.  Table 2 summarized the *L*_Aeq_ of 22 different noise samples and OHC and IHC (at six different one-octave bands) of animals in each group corresponding to the noise sample.

## 3. Results and Discussions

### 3.1. BM Velocities Distribution in Chinchilla Cochlea

#### 3.1.1. BM Velocities Distribution Obtained by the TRNL Filter

**Figure 5:**
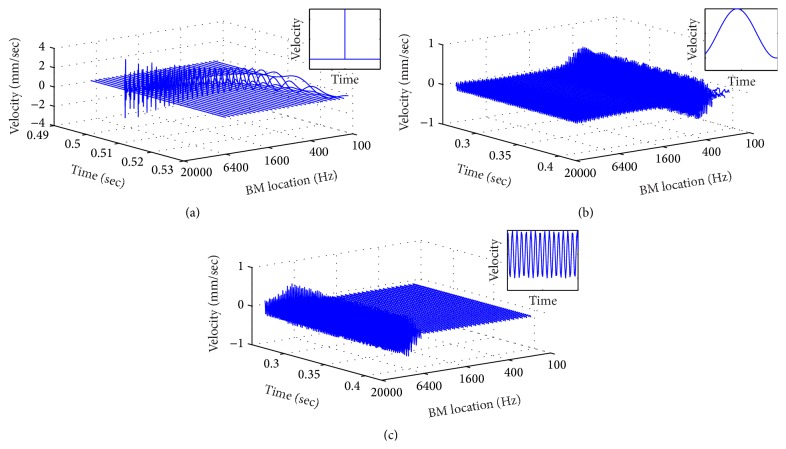
Time-frequency presentations of BM velocities as the output of the TRNL filter, responding to (a) impulsive noise, (b) sweeping chirp noise at low frequency (400–500 Hz), and (c) sweeping chirp noise at high frequency (8000–12000 Hz). The labels of frequency axis indicate the different locations along BM, which refer to the partitions in cochlea.

The distribution of BM velocities is obtained by the TRNL filter (as shown in Figure 3). In this section, two noise signals, referred as impulse noise and sweeping chirp noise, were simulated and fed into the TRNL filter to demonstrate the response of BM in the cochlea [38].  Figure 5 shows the time-frequency (*T*-*F*) distributions of the BM velocities in the cochlea as the output of the TRNL filter, responding to the simulated noises. The frequency axis labeled as the locations along the BM, which refers to the partitions in the cochlea. Each partition works as a bandpass filter, which can be represented by the ERBs with different CFs. The highest CF is at the base of the cochlea, and CFs decrease from the base to the apex of the cochlea [17].

BM velocity responding to the simulated impulse noise (the Dirac delta function) is shown in Figure 5(a). Along with the BM, from base to apex of the cochlea (high frequency ranges to low frequency ranges), the amplitudes of BM velocities are decreasing and the pulse duration of the velocity waveform becomes longer. The amplitudes of BM velocities at different locations can reflect the gains of local filters in the cochlea. Results indicate that the TRNL filter can accurately simulate the motion of the BM in chinchilla's cochlea.

Furthermore, two chirp noise signals in different frequency bands were simulated and applied to the TRNL filter to validate the BM motion responding to different frequency components. Figures 5(b) and 5(c) show the BM velocities responding to sweep chirp signals at low (400–500 Hz) and high frequency bands (8000–12000 Hz), respectively. It can be found that the distribution of BM velocities correlated with the input signals very well. The BM velocities concentrate at the low frequency range (400–500 Hz) as response to the low frequency signal in Figure 5(b). In contrast, the BM velocities focus at high frequency range (8000–12000 Hz) as response to the high frequency signal in Figure 5(c). The results demonstrate that the TRNL filter accurately catches both time and frequency features of the simulated signals.

#### 3.1.2. BM Velocities Distribution Obtained by the Chinchilla Auditory Model

**Figure 6:**
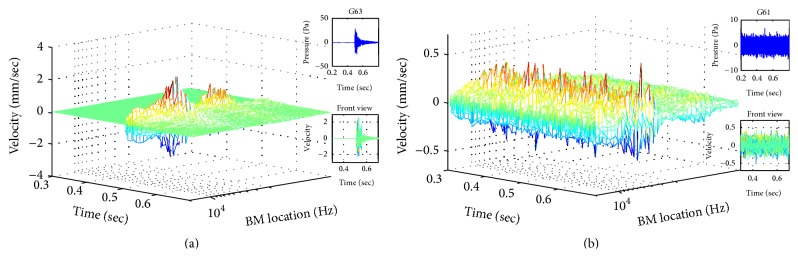
Time-frequency presentations of the BM velocities obtained by the developed chinchilla auditory model, responding to two experimental noise samples: (a) G63 and (b) G61. The partial waveforms of G63 and G61 in 0.5 sec are shown in the top insert figures. The front views of the distributions of the BM velocities are shown in the bottom insert figures.

Two experimental noise samples (i.e., G63 and G61) were used as inputs to validate the developed chinchilla auditory model, including the consecutive external-middle ear and the inner ear.  Figure 6 shows the BM velocities distribution in the *T*-*F* domain as the output of the developed chinchilla auditory model. The noise sample G63, as shown in the insert figure in Figure 6(a), simulates a local impulsive noise, while the sample G61, as shown in the insert figure in Figure 6(b), is a typical Gaussian continuous noise.

As shown in the front views in Figures 6(a) and 6(b), the distributions of the BM velocities along the time axis can accurately reflect the waveform of the original noise signals. It indicates that the developed chinchilla auditory model works well on transferring the acoustic pressure to the BM velocities. In addition, along the frequency axis, the distributions of the BM velocities are concentrated in the high frequency bands. The BM velocities in the low frequency bands are significantly reduced. This is caused by the gain of transfer function of the external ear of chinchilla (as shown in Figure 2(a)), which demonstrates a strong decayed gain at low frequency range.

As shown in Figures 5 and 6, the BM velocities can be both positive and negative, which reflect the direction of movements of BM. When BM vibrates up and down, its movements cause stretching and squeezing against hair cells in cochlea, respectively. Both stretching and squeezing motions could damage hair cells.

### 3.2. Validation of the Developed EVL and CVL Fatigue Models Using Animal Data

The correlations of the developed fatigue metrics (i.e., *L*_EVL_ and *L*_CVL_) and NIHL (hearing loss indices) can be evaluated by applying the linear regression analysis as described in [57]. A single-variable regression analysis form is used as an for example,

(8) $$\begin{array}{c} {\text{PTS}}_{\text{Loss}}=c_{0}+c_{1}L_{metric}+\epsilon , \end{array}$$

where *ϵ* is the error to be minimized and *L*_metric_ refers to the proposed fatigue metrics. The expressions of other three hearing loss indices are the same as PTS_Loss_ in (8).

#### 3.2.1. Linear Regression Analysis at Six One-Octave Bands

**Figure 7:**
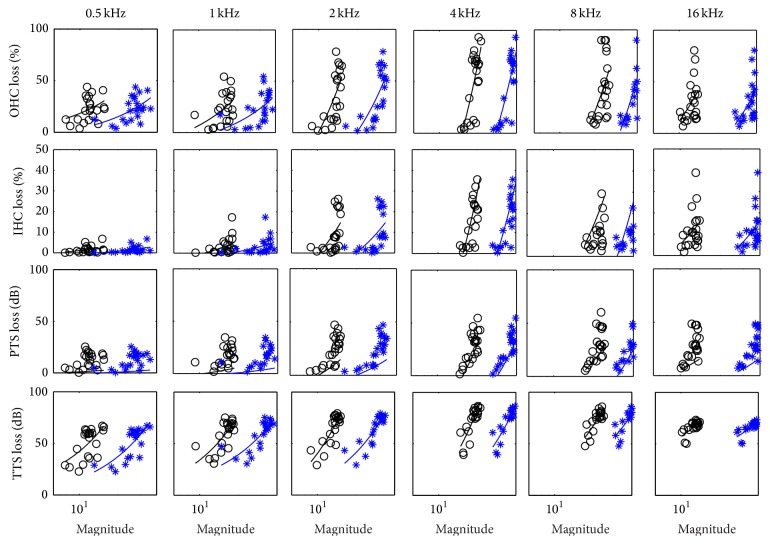
Scattering plots and fitting lines of pairs of the developed fatigue metrics, *L*_EVL_ (black color) and *L*_CVL_ (blue color), and four hearing loss indications (i.e., OHC loss, IHC loss, TTS, and PTS) at six one-octave frequency bands, averaged by all 22 groups of animal experimental data. The *p* values have been summarized in Table 3.

The linear regression analysis of the two developed fatigue metrics and four hearing loss indications (i.e., OHC loss, IHC loss, PTS, and TTS) at six one-octave frequency bands has been conducted using all 22 groups of experimental data.  Figure 7 shows the fitting lines and scattering plots of the pairs of the metrics and the hearing loss indices. Both *L*_EVL_ and *L*_CVL_ are calculated using a 40 sec time window. Each symbol in Figure 7 refers to a pair of a fatigue metric and an animal hearing loss index. The lines indicate the fitting results of the distributions of symbols. Six one-octave frequency bands centered at 0.5, 1, 2, 4, 8, and 16 kHz cover the whole frequency range of the BM. It can be found in Figure 7 that the magnitudes of both metrics and amplitudes of all four hearing loss indices in the high frequency bands (2, 4, and 8 kHz) are larger than the corresponding values in the low frequency bands (0.5 and 1 kHz). In addition, as shown in Figure 6, the amplitudes of BM velocities in the high frequency bands are greater than that in the low frequency bands. The higher BM velocity reflects stronger vibration of the BM, which eventually lead to more hearing loss in the cochlea. The results indicate that both EVL and CVL models can reflect the BM motion stimulated by noise and accurately predict noise induced hearing loss in chinchilla.

Moreover, the *r* ^2^ value is used to reveal the linear correlation between two variables. *r* ^2^ = 1 indicates a perfect correlation and *r* ^2^ = 0 indicates no correlation between the fatigue models and NIHL. The values of *r* ^2^ are summarized in Table 3. In Table 4, the *r* ^2^ values at 2, 4, and 8 kHz are higher than these values at 0.5 and 1 kHz. It means that both *L*_EVL_ and *L*_CVL_ have strong correlations with hearing loss indices at frequency bands centered at 2, 4, and 8 kHz. In contrast, the metrics demonstrate weak correlations with hearing loss indices at low frequency bands, for example, 0.5 and 1 kHz. The results are consistant with Figure 7. In addition, both *L*_EVL_ and *L*_CVL_ show the highest correlations with TTS than other three hearing loss indices (i.e., IHC loss, OHC loss, and PTS) at all frequency bands. TTS refers to the instant hearing loss immediately after a noise exposure, while the PTS is the permanent hearing loss after noise exposure and certain recovery time [33]. TTS directly reflects the mechanical damage caused by noise exposure. The developed fatigue models are based on the BM velocity, which reflects the mechanical vibration of BM in cochlea. Therefore, it is reasonable that the proposed fatigue metrics have highest correlations with TTS. One can also find that OHC loss has higher correlation with fatigue metrics than IHC loss. It indicates that OHC loss has more association with a mechanical fatigue phenomenon than IHC loss. As shown in Table 3, most of *p* values of linear regressions are lower than 0.05. There are eight *p* values higher than 0.05, including *L*_EVL_-OHC at 0.5 kHz, *L*_EVL_-IHC at 0.5, 1, 8, and 16 kHz, *L*_CVL_-IHC at 0.5 and 1 kHz, and *L*_EVL_-PTS at 0.5 kHz. The results indicate that most of the linear regressions are statistically significant.

#### 3.2.2. Linear Regression Analysis at Averaged Frequency Bands

**Figure 8:**
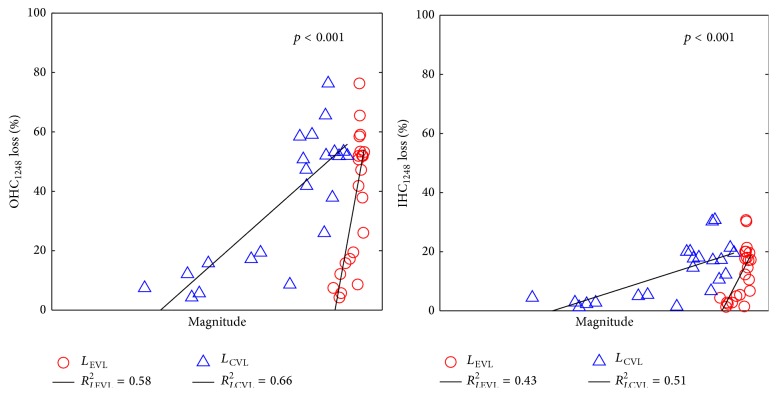
Regression analysis of averaged OHC_1248_ and IHC_1248_ loss and mammal auditory system based fatigue metrics.

**Figure 9:**
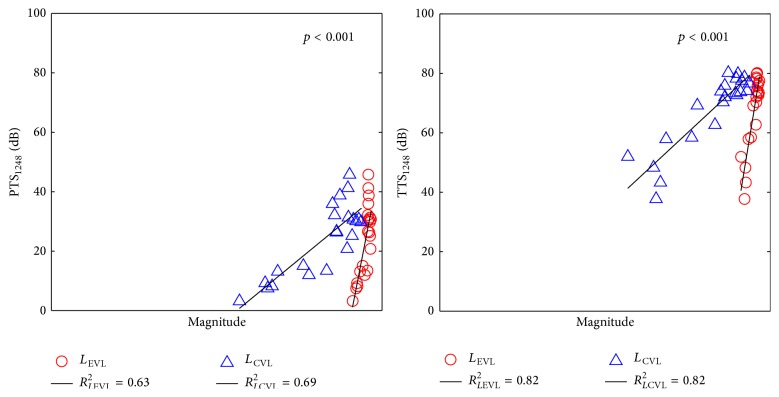
Regression analysis of averaged PTS_1248_ and TTS_1248_ and mammal auditory system based fatigue models.

To further evaluate the effectiveness of the proposed fatigue metrics on NIHL prediction, hearing loss indices averaged by the one-octave bands centered at 1, 2, 4, and 8 kHz, including IHC_1248_ loss, OHC_1248_ loss, TTS_1248_, and PTS_1248_, were used for the regression analysis.  Figure 8 shows the scattering plots and the fitting lines of the pairs of the developed fatigue metrics and IHC_1248_ loss and OHC_1248_ loss. It can be found that OHC_1248_ loss is larger than IHC_1248_ loss, and both *L*_EVL_ and *L*_CVL_ have stronger correlations with OHC_1248_ loss than with IHC_1248_ loss. The scattering plots and the fitting lines of the pairs of the developed fatigue metrics and TTS_1248_ and PTS_1248_ are shown in Figure 9. It can be found that both metrics demonstrated stronger correlation with TTS_1248_ than with PTS_1248_.

The regression analysis results of the fatigue models and four effective hearing loss indices are summarized in Table 4. It confirms the observations in Figures 8 and 9. Overall, both EVL and CVL models achieved high *r* ^2^ values with all four effective hearing loss indices. This indicates that both developed fatigue models have high correlations with the chinchilla experimental hearing loss data. Specifically, the *r* ^2^ values between the fatigue metrics and two major hearing loss indices (TTS_1248_ and PTS_1248_) are higher than 0.65, which is considerable high value in the regression analysis. Further, all the *p* values are lower than 0.001; consequentially the linear regressions are statistically significant. It means that the developed fatigue models can accurately predict NIHL in chinchilla.

Moreover, one can also see that, in both Tables 3 and 4, the CVL model has higher correlation with all four hearing loss indices than the EVL model. One of the potential explanations may be that most of the experimental noise samples used in this study are complex noise with intense transitions. The EVL model treats such kind of complex loads as the summation of independent sinusoid loads (i.e., pure tone) in frequency domain and neglects the strong transitions of adjacent stimulus in the time domain. It may underestimate the hearing loss caused by the complex noise with high kurtosis value. In contrast, the CVL model is designed based on Miner's rule, and it incorporates calculations on the hearing loss caused by transitions of the complex loads. Therefore, the analysis results demonstrate that the CVL model can more accurately predict the hearing loss caused by highly transited complex noise than the EVL model. For low kurtosis noise, or steady-state noise, transitions of adjacent loads are comparable with pure tone. Accordingly, both EVL and CVL models can be applied to predict GDHL.

## 4. Conclusion

In this study, we developed two fatigue models (i.e., the EVL and CVL models), which combined the high-cycle fatigue model with the mammalian auditory model, to predict GDHL. The high-cycle fatigue theory was used because GDHL caused by occupational noise can be considered as a long-time process of physical compression and stretching of organ of Corti. The mammalian auditory model was introduced by combining the TRNL filter with the transfer function of the external-middle ear to accurately characterize the vibration of BM. A series of animal noise exposure data were used to validate the effectiveness of the developed fatigue models. The regression analysis of fatigue models and four hearing loss indices was conducted. Results showed that both fatigue models have high correlations with animal hearing loss data. It indicates that the developed models can accurately predict the NIHL in chinchilla. In addition, the CVL model demonstrated higher correlations with four hearing loss indices than the EVL model. The CVL model would be more accurate on the evaluation of the auditory risk of exposure to hazardous occupational noise. In our future work, we will develop fatigue based models for prediction of auditory risk on human exposed to high-level occupational noise.

## Figures and Tables

**Table 1:** Parameters of the TRNL algorithm used for the simulation of chinchilla's inner ear [28].

	800 Hz	5500 Hz	7250 Hz	9750 Hz	10000 Hz	12000 Hz	14000 Hz
Linear							
Gammatone cascade	5	5	5	5	5	5	5
Low pass cascade	7	7	7	7	7	7	7
CF_lin⁡_	750	5000	7400	9000	9000	11000	13000
BW_lin⁡_	450	3000	2500	3000	3500	5000	4000
LP_lin⁡_	750	6000	7400	9000	8800	12000	13500
Gain, *g*	500	190	3000	300	500	500	350
Nonlinear							
Gammatone cascade	3	3	3	3	3	3	3
Low pass cascade	4	4	4	4	4	4	4
CF_lin⁡_	730	5850	7800	9800	10000	12000	15000
BW_lin⁡_	350	1800	2275	1650	1800	2000	3200
LP_nl_	730	5850	7800	9800	10000	12000	15000
Gain, *a*	850	3000	15000	9000	15000	22500	3000
Gain, *b*	0.03	0.04	0.06	0.05	0.06	0.07	0.045
Exponent, *c*	0.25	0.25	0.25	0.25	0.25	0.25	0.25
Linear all-pass							
Gain, *k*	10	0.4	20	1	2	20	20

**Table 2:** OHC and IHC loss at different center frequencies of one-octave bands for different noise exposures.

Samples	*L*_Aeq_	OHC						IHC					
		0.5 kHz	1 kHz	2 kHz	4 kHz	8 kHz	16 kHz	0.5 kHz	1 kHz	2 kHz	4 kHz	8 kHz	16 kHz
G44	100.6	20.8	38.1	67.9	67.5	62.7	42.7	1.6	5.6	22.8	21	22.7	6.9
G49	101	43.8	54.6	78.3	93.1	79.3	71.7	2.1	4.6	24.8	63.9	29.5	26.7
G50	100.5	11.8	21.7	15	12.7	28	25.6	0.7	2.6	2	4.7	12.3	10.7
G51	100.1	32.6	30.9	43.9	49.7	27	13.8	3.1	6.6	9.6	21.2	5	4
G52	101.7	39.1	40.8	64.7	66.3	41	20.5	3.4	6.8	18.8	35.6	7.6	6.9
G53	100.6	27.8	39.3	50.7	67.6	49.9	32.1	1	4	7.5	23.3	14.2	5
G54	100.6	21	23.7	60.4	69.9	35.1	17.8	1.1	1.8	22.1	22.7	11.9	9.4
G55	100.1	40.7	36.4	55.5	80.4	89.9	80.5	6.7	9.7	7.8	28.3	75.2	38.9
G60	100.2	35	34.2	54.1	72.4	47.5	29.5	2.1	2.4	22.6	32.1	11.3	12.3
G61	99.6	7.9	5.6	4.6	9.9	14.3	17.2	0.2	0.2	0.2	2.8	2.6	8.1
G63	99.6	35.6	50.1	65.8	75.6	42.3	36.1	5.1	17.3	26.2	26.2	10.2	11
G64	101.1	15.6	11.3	25.7	50.8	16	14.5	0.6	0.6	3.4	16.7	6.1	9.1
G65	99.7	24	16.7	25.1	71.3	90	46.7	1.5	1.8	1.4	15.4	61.6	22.9
G66	100.7	21.3	11.2	12.9	60.5	82.7	37.9	0.8	1	0.6	12.9	56.2	16.5
G68	99.7	22.8	22.4	30.5	70.4	89.9	58.9	1	1.6	7.7	23.8	52.3	15.9
G69	101	12.6	17.1	11.6	7.4	12.6	11.6	0.1	0.2	2.7	2.8	5.5	4.5
G70	100.7	23.4	19.9	52.2	89.6	46.1	38	0.7	0.9	8.8	50.7	18	16.4
G47	89.4	3.8	2.8	6.2	3.7	17	20.7	0.3	2.2	2.8	3.9	8.9	4
G48	91.7	5.9	4.6	2.5	5.8	9.7	15	0.4	0.4	1.5	2.7	4.6	9.5
G56	91.3	9	3.7	1.9	3.3	8.3	6.9	0.3	0.7	0.7	0.4	3.3	1.7
G57	94.2	28	24.2	15.7	10.2	13	16.7	0.4	0.6	2.5	3.2	5	11.6
G58	95.6	12.3	5.6	12.9	34.1	16.1	12.4	1	0.6	2.6	11	5.8	4.8

**Table 3:** Regression analysis at six one-octave bands centered at 0.5, 1, 2, 4, 8, and 16 kHz.

*r* ^2^/*p*	0.5 kHz	1 kHz	2 kHz	4 kHz	8 kHz	16 kHz
*L*_EVL_-OHC	0.22/0.08	0.18/0.04	0.48/<0.001	0.56/<0.001	0.28/0.01	0.18/0.05
*L*_CVL_-OHC	0.26/0.01	0.26/0.01	0.69/<0.001	0.71/<0.001	0.48/<0.001	0.27/0.01
*L*_EVL_-IHC	0.21/0.16	0.16/0.07	0.56/0.007	0.47/<0.001	0.14/0.08	0.24/0.12
*L*_CVL_-IHC	0.25/0.06	0.20/0.06	0.54/0.01	0.56/<0.001	0.22/0.02	0.27/0.04
*L*_EVL_-PTS	0.13/0.11	0.28/0.009	0.52/<0.001	0.58/<0.001	0.40/0.002	0.57/0.001
*L*_CVL_-PTS	0.25/0.01	0.41/0.001	0.77/<0.001	0.71/<0.001	0.52/<0.001	0.65/0.001
*L*_EVL_-TTS	0.39/0.003	0.53/<0.001	0.70/<0.001	0.71/<0.001	0.68/<0.001	0.58/<0.001
*L*_CVL_-TTS	0.57/<0.001	0.65/<0.001	0.81/<0.001	0.76/<0.001	0.72/<0.001	0.79/<0.001

**Table 4:** Regression analysis at spectrum [1 kHz–8 kHz].

Pair	*r*_2_	*p*
*L*_EVL_-OHC_1248_	0.59	3.4 × 10^−5^
*L*_CVL_-OHC_1248_	0.66	4.7 × 10^−6^
*L*_EVL_-IHC_1248_	0.43	9.5 × 10^−4^
*L*_CVL_-IHC_1248_	0.51	2.0 × 10^−4^
*L*_EVL_-PTS_1248_	0.63	1.0 × 10^−5^
*L*_CVL_-PTS_1248_	0.69	1.6 × 10^−6^
*L*_EVL_-TTS_1248_	0.82	5.8 × 10^−9^
*L*_CVL_-TTS_1248_	0.82	7.4 × 10^−9^
